# Resource partitioning confirmed by isotopic signatures allows small mammals to share seasonally flooded meadows

**DOI:** 10.1002/ece3.5144

**Published:** 2019-04-08

**Authors:** Linas Balčiauskas, Raminta Skipitytė, Laima Balčiauskienė, Marius Jasiulionis

**Affiliations:** ^1^ Nature Research Centre Vilnius Lithuania; ^2^ Centre for Physical Sciences and Technology Vilnius Lithuania

**Keywords:** dietary separation, diversity, isotopic partitioning, small mammal community, spring floods

## Abstract

Meadows in river deltas are characterized by a high diversity and abundance of small mammals. However, neither their spatial arrangement nor differences in their use of microhabitat can necessarily explain the dense co‐occurrence of sympatric species. We investigated how several small mammal species share a seasonally flooded meadow of limited size, testing predictions (P1) that herbivore, granivore, insectivore, and omnivore species are separated in time (dominant in different years), (P2) that sympatric species undergo isotopic partitioning, and (P3) that there are intraspecific differences in diet. Stable carbon and nitrogen isotope signatures in the hair of seven synantropic shrew, vole, and mice species were used as a proxy for their diet. We found that the three most abundant species in eight of the nine years were from different diet groups. However, based on the number of species in the functional groups, the state of small mammal community was considered unfavored in five out of the nine investigation years. In years with the greatest dominance of *Apodemus agrarius*, the small mammal community was characterized by decreased diversity and *Micromys minutus* was either in low abundance or absent. In 2014 and 2016, years of low abundance or absence of *M. oeconomus*, *M. agrestis,* and *M. glareolus* were both recorded in high numbers. Differences in the isotopic signatures of the three most abundant small mammal species in the community were clearly expressed and core areas in the isotopic space were separated, showing their dependence on different dietary resources. Intraspecific dietary separation between young and adult animals was observed only in *M. oeconomus*. Thus, the high species diversity of small mammals and the formation of their community in this investigated flooded meadow are maintained by isotopic partitioning (segregation in dietary space) and by changes in their number over time (shifting dominance).

## 1 INTRODUCTION

Co‐occurring species inevitably will compete for space and resources, and this competition changes their distribution (Baltensperger, Huettmann, Hagelin, & Welker, 2015). While dense co‐occurrence of sympatric species may be attributed to differences in microhabitat use (Jorgensen, 2004), not all cases can be explained by this “microhabitat paradigm” (Balestrieri et al., 2017). Alternative means to coexist can be through a spatial arrangement of species (Myllymäki, 1977; Wilson et al., 2014), differing diets (Shiels et al., 2013), or via dietary separation of species with similar requirements, that is, resource partitioning (Calandra et al., 2015; Dueser & Shuggart, 1979; Meserve, 1981; Schoener, 1974; Symes, Wilson, Woodborne, Shaikh, & Scantlebury, 2013). Additional drivers may also influence the temporal and spatial placement of resources and small mammals (Balestrieri et al., 2017; Marques, Rocha, Mendes, Fonseca, & Ferreira, 2015; Sozio & Mortelliti, 2016).

River floodplains, affected by periodic floods, are productive and heterogeneous habitats (Mathar, Kleinebecker, & Hölzel, 2015), suitable for small mammals (Wijnhoven, Smits, Van der Velde, & Leuven, 2006). After recovery from the detrimental influences of floods, the abundance of small mammals in river floodplains is high for a given period of time (Golet, Hunt, & Koenig, 2013).

Areas subject to periodic flooding maintain a high small mammal diversity (Balčiauskas, Balčiauskienė, & Janonytė, 2012b; Crnobrnja‐Isailović et al., 2015) as the dynamic hydrology supports a diversity of resources (Merwe & Hellgren, 2016). A greater number of species (Barnosky, Hadly, Maurer, & Christie, 2001; Hallett, 1991) or functional diversity of these species (Wood, McKinney, & Loftin, 2017) enhances the stability of a community, increasing the potential to withstand negative influences (Scheffer et al., 2012). Under conditions of increased and more frequent floods (prognosis by Reader, Stedmon, & Kritzberg, 2014), ecosystems may reorganize (Brown, Whitham, Ernest, & Gehring, 2001). The arrival of new species and resulting changes in food webs (Baltensperger et al., 2015) may be buffered by compensation from complementary species.

Rodent species are characterized by different diets, and thus, the isotopic niche of this complex taxonomic group is broad (Galetti, Rodarte, Neves, Moreira, & Costa‐Pereira, 2016). The diets of voles and mice partially overlap, but fundamentally differ from other small mammals such as marsupials and shrews (Baltensperger et al., 2015; Butet & Delettre, 2011; Galetti et al., 2016; Symes et al., 2013). In small rodent communities from transitional temperate climates, three groups are recognized according to their diet, specifically herbivorous voles (*Microtus* and *Arvicola*), granivorous mice (*Apodemus* and *Micromys*), and the omnivorous *M. glareolus* (Butet & Delettre, 2011; Zub, Jędrzejewska, Jędrzejewski, & Bartoń, 2012). All shrews (*Sorex* and *Neomys*) in the temperate region are insectivorous, with the species utilizing dietary separation and microhabitat selection to allow them to coexist in the same habitat (Churchfield & Rychlik, 2006; Pernetta, 1976).

Diet differences may favor coexistence in sympatric species (Kronfeld‐Schor & Dayan, 1999; Luo & Fox, 1996; Shiels et al., 2013) and are also characteristic of other systematic groups of mammals, for example, carnivores (Kasper, Peters, Christoff, & de Freitas, 2016).

Small mammal communities are not randomly assembled, they follow so‐called “assembly rule” (Fox & Kirkland, 1992). It says that “each species entering a community will tend to be drawn from a different group until each group is represented, and then the rule repeats” (Fox & Brown, 1993). According to Fox (1987), we should expect a single species from each of the different dietary groups to form the community in years with low small mammal diversity, with increasing resources thereafter allowing the addition of a second species from each group, then a third, ultimately resulting in a favorable community structure (see Data analysis). As these small mammal groups reflect dietary separation, isotopic partitioning also should be expected (Calandra et al., 2015; Hwang, Millar, & Longstaffe, 2007).

The aim of the study was to investigate the pattern of coexistence of several small mammal species in a seasonally flooded meadow, based on the working hypothesis that, in order to coexist in a small area, species should be separated not only in dietary space but also in time. We supposed that separation in dietary space would operate for a single year, while shifting dominance would operate over the longer periods, this additionally reflecting the differing resilience of various species to floods. We tested three predictions: P1—sympatric species of the same group (herbivores, granivores, insectivores, and omnivores) are separated by time, that is, dominate in different years, P2—sympatric species are separated in dietary space, thus differ in isotopic signatures, and P3—intraspecific differences between various demographic groups are present (assuming intraspecific competition for food). P3 is based on our previous and ongoing research (Balčiauskas, Skipitytė, Jasiulionis, Balčiauskienė, & Remeikis, 2018; Balčiauskas et al., 2016), where we found some intraspecific segregation in the isotopic space in yellow‐necked mice (*Apodemus flavicollis*) and bank voles (*Myodes glareolus*) living in great cormorant colonies, an environment where foods are scarce, thus necessitating competition.

## 2 MATERIAL AND METHODS

### 2.1 STUDY SITE

**Figure 1:**
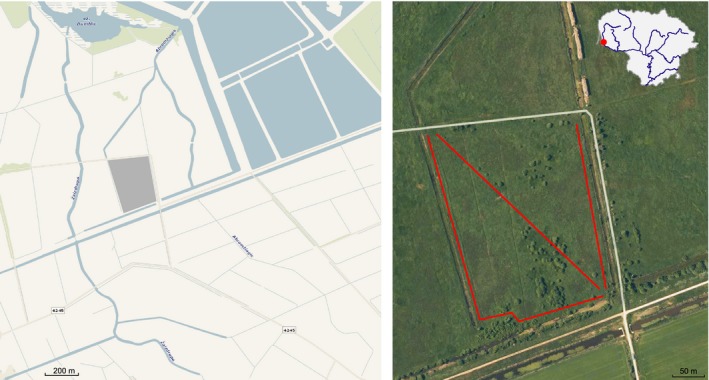
Study site position in the Nemunas River Delta (between Nemunas (Atmata) and Skirvytė river branches) and habitat structure of the site. Red lines represent trap setting lines in 2008–2016. The diagonal line was operational in 2009 only

We studied the small mammal community of a flooded meadow (55°19'26.23"N, 21°20'24.15"E) near Rusnė settlement (55°20'10''N; 21°18'54''E) in the Nemunas River Delta, situated in western Lithuania (Figure 1). The delta is on the border of two major biogeographical regions in Europe (European Environment Agency, 2002), namely the boreal and continental, and thus, the small mammal community encompasses species from both.

The area of the site is quite small (7.05 ha, with a perimeter of 1,070 m) and is flooded every year (Balčiauskas et al., 2012b), with the duration of submergence dependent on flood height. Regardless of flood level, the trapping site is totally flooded for only a short time each spring. Spring floods normally start around 19 of March, and the average duration of flood is 16 days (Floods, 2018). In the study area, spring floods effectively eradicate the small mammal communities in the meadow, but the negative effects are short‐term and high small mammal diversities are restored during the summer period from enclosing levees, serving as refugees during flood (Balčiauskas et al., 2012b).

The area consists of a polder system with artificially raised embankments to protect against high spring floods. The meadows are surrounded by ditches, overgrown by reeds and partially by shrubs (Figure 1). The main vegetation of the meadow consists of Poaceae and Cyperaceae plants. These flooded meadows were not cut during the investigation period, except in 2012–2013 when vegetation from the central part of the site was cut once during the summer of each year, though the surrounding reed belts were left untouched. Trapping was performed at a time when the cut surface had re‐grown. Visual assessment of the habitat and measuring several variables, such as grass height, reedbed presence, shrub presence, distance to the water, main species of the vegetation at all of the trapping locations in 2011–2016, confirmed its uniformity (*unpublished data*).

### 2.2 Small mammal trapping

Small mammals were trapped in 2008–2016. In 2011 and 2013**–**2016, trapping occurred once at the end of September/beginning of October. In the other years, there were two or three trapping sessions (July–September). In the years with several trapping sessions, there were no shifts in the numbers of the two most numerous small mammal species between the trapping sessions, so the data were pooled. Each year, we used 6–31 lines of 25 snap traps, each set 5 m apart, the number of lines depending on the number of trapping sessions (Table 1). We positioned the traps according to the perimeter of the site in all years, the trap lines being close to drainage ditches (2–10 m) and adjacent reed belts. In 2009, traps were additionally set on a diagonal transect (Figure 1). Traps were set for three days, checked once a day, and baited with bread crust and sunflower oil. The total trapping effort was 7,651 trap nights, and 1,359 individuals of 11 species were captured (Table 1). Presented in the Supporting information Table S1, relative abundance was expressed as standard capture rates to number of animals/100 trap nights. Most of the registered species were typical for the region. In Lithuania, common vole (*Microtus arvalis*)*,* common shrew (*Sorex araneus*), *M. glareolus*, *A. flavicollis*, striped field mouse (*Apodemus agrarius*), and pygmy shrew (*Sorex minutus*) are typical meadow species.

**Table 1:** Composition of the small mammal community in a seasonally flooded meadow at Rusnė (western Lithuania), 2008–2016 and trapping effort

Species	2008	2009	2010	2011	2012	2013	2014	2015	2016	Total	
										*N*	%
*Sorex araneus* ^a^	35	42	31	2	44	9	14	9	7	193	14.2
*Sorex minutus* ^a^	3	8	12	–	3	2	–	4	3	35	2.6
*Neomys fodiens* ^a^	2	–	–	–	–	–	–	–	–	2	0.1
*Apodemus agrarius* ^b^	22	60	193	124	57	17	55	53	63	644	47.4
*Apodemus flavicollis* ^b^	–	–	–	–	3	–	–	–	–	3	0.2
*Micromys minutus* ^b^	53	–	33	–	10	–	5	2	1	104	7.7
*Microtus arvalis* ^c^	–	2	–	–	–	–	2	–	–	4	0.3
*Microtus agrestis* ^c^	–	–	1	–	–	–	3	10	7	21	1.5
*Microtus oeconomus* ^c^	46	102	30	14	67	5	7	37	–	308	22.7
*Myodes glareolus* ^d^	–	–	13	2	2	1	10	13	3	44	3.2
*Arvicola amphibius* ^c^	–	–	1	–	–	–	–	–	–	1	0.1
Total, *N*	161	214	314	142	186	34	96	128	84	1,359	100
No of species	6	5	8	4	7	5	7	7	6	11	
Shannon's *H*	2.10	1.73	1.85	0.67	2.03	1.80	1.98	2.19	1.33	2.13	
Simpson's *c*	0.26	0.35	0.41	0.77	0.28	0.35	0.37	0.28	0.58	0.30	
Trapping effort, trap lines	20	31	23	8	12	6	9	6	6	121	
Trapping effort, trap nights	750	1995	1525	600	750	450	681	450	450	7,651	

Notes

Diet preferences marked with superscripts: a—insectivores, b—granivores, c—herbivores, d—omnivores (according to Butet & Delettre, 2011; Churchfield & Rychlik, 2006; Zub et al., 2012; Pernetta, 1976). Shannon's H measures diversity of the small mammal community, Simpson's c the dominance. Trapping effort is expressed in trap nights

Species were identified morphologically, with specimens of *Microtus* voles identified by their teeth. Juveniles, subadults, and adults were identified under dissection, based on body weight, the status of sex organs and atrophy of the thymus, the latter of which decreases with animal age (Balčiauskas, Balčiauskienė, & Janonytė, 2012a). After cleaning using *Dermestes* beetles, skulls were deposited at the Laboratory of Mammalian Ecology of the Nature Research Centre (Vilnius, Lithuania).

### 2.3 Stable isotope analysis

To test predictions P2 and P3, hair samples were collected in 2015 from 81 individuals of the seven small mammal species for stable isotope analysis (Table 2). We clipped off a tuft of hair from between the shoulders of each specimen and stored it dry in separate bags. Scissored samples were weighed with a microbalance and packed in tin capsules, and stable isotope analysis was then carried out. Carbon and nitrogen stable isotope ratios were measured using an elemental analyzer (EA) (Flash EA1112) coupled to an isotope ratio mass spectrometer (IRMS) (Thermo Delta V Advantage) via a ConFlo III interface (EA‐IRMS).

**Table 2:** Small mammal samples used for stable isotope analysis from a seasonally flooded meadow at Rusnė, 2015 (animal age and sex in insectivores not always known due to self‐digestion* of the internal organs)

Species	*N*	Males	Females	Adults	Subadults	Juveniles
*Sorex araneus*	5	2	2	–	1	–
*Sorex minutus*	3	–	–	–	–	–
*Apodemus agrarius*	12	8	4	1	5	6
*Micromys minutus*	1	–	1	–	–	1
*Microtus agrestis*	11	2	9	3	3	5
*Microtus oeconomus*	34	14	20	15	7	12
*Myodes glareolus*	15	10	5	2	2	11

* In shrews after trapping with snap traps, digestion processes do not stop, thus resulting in abdominal organs, including testes, uterus, and ovaries, being unavailable for sex determination. Self‐digestion of *gl. thymus* does not allow for age estimation of an individual. For the other species, self‐digestion is not characteristic.

Carbon and nitrogen isotope data are reported as *δ* X values (where X represents the heavier isotope ^13^C or ^15^N) or differences from given standards, expressed in parts per thousand (‰), and are calculated according to the formula:

$$\delta X=[R_{\text{sample}}/R_{\text{standard}}-1]\times 1000.$$

where *R*_sample_ = ^13^C/^12^C or ^15^N/^14^N of the sample, *R*_standard_ = ^13^C/^12^C or ^15^N/^14^N of the standard.

Reference materials Caffeine IAEA‐600 (*δ* ^13^C = −27.771 ± 0.043‰, *δ* ^15^N = 1 ± 0.2‰) and oil NBS‐22 IAEA (*δ* ^13^C = −30.031 ± 0.043‰) provided by the International Atomic Energy Agency (IAEA) were used as standards for calibration of the reference gases (CO_2_ and N_2_). EMA P2 (Elemental Microanalysis, *δ* ^13^C = −28 ± 0.1‰, *δ* ^15^N = −2 ± 0.2‰) was selected as a laboratory working standard. Repeated analysis of this reference material gave a standard deviation of less than 0.08‰ for carbon and 0.2‰ for nitrogen (Balčiauskas et al., 2016).

### 2.4 Data analysis

The diversity of the small mammal community was expressed using the Shannon–Wiener diversity index, H, on the base of log_2_ (Krebs, 1999), while dominance was expressed using the Simpson's index c (Golet et al., 2013; Krebs, 1999; Zhang et al., 2007). Diversity of the community was compared to other habitats and territories of different size in Lithuania, data from Balčiauskas and Juškaitis (1997).

We checked if there was a correlation between diversity and *A. agrarius* dominance (this a generally uncommon species in the country, but strongly dominant during most of the investigation). Dominance was calculated as a percentage of the total number of trapped individuals. Pearson's *r* was used as dominance values were distributed normally.

**Figure 2:**
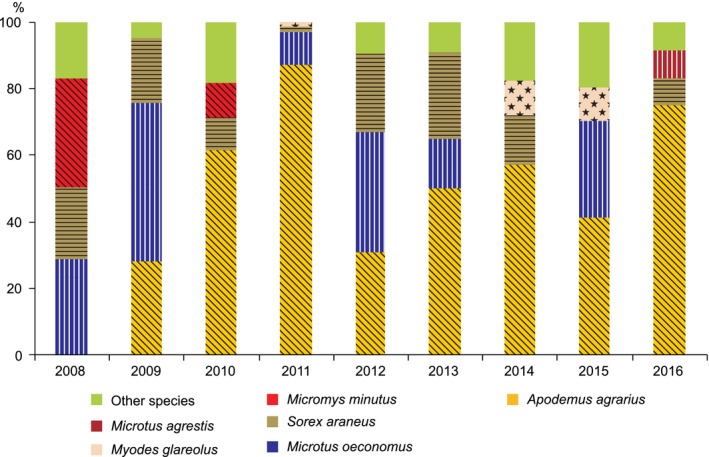
Temporal changes in the numbers of small mammal species in the Rusnė flooded meadow (less numerous species pooled). Dietary groups indicated by hatching (vertical—herbivores, diagonal—granivores, horizontal—insectivores) and pattern (stars—omnivores)

Prediction P1 was tested according to the rule of equal representation of functional groups (insectivores, granivores, herbivores, and omnivores) in a small mammal community (Fox, 1987). Accordingly, if the difference between the numbers of species trapped in these four groups in any year is >1, the state of the community is considered unfavorable. The distribution of favored and unfavored states of the small mammal communities in the Rusnė flooded meadow is presented in Supporting information Table S2. The pool of species in the area was insectivores (*I*) = 3, granivores (*G*) = 3, herbivores (*H*) = 4, and omnivores (*O*) = 1. Consequently, the probability of their presence in the community was *I* = 0.273, *G* = 0.273, *H* = 0.364, and *O* = 0.090, respectively. We calculated the expected number of species in the functional groups for every year of the investigation. The significance between expected and observed numbers was tested using a chi‐square test. Representation of the functional groups in the community was also evaluated using the three most abundant species in any year (Figure 2).

The *δ* ^13^C and *δ* ^15^N values in the samples were expressed as arithmetic mean ± 1 *SE*. Normality of the *δ* ^15^N and *δ* ^13^C values was evaluated using Kolmogorov–Smirnov test. Based on conformity to normal distribution, parametric tests were used. Main‐effects ANOVA was used to find the relationship of dietary group, species, age, and sex of individuals to paired *δ* ^15^N and *δ* ^13^C distribution, using Hotelling's two sample *T* ^2^ test for significance.

The influences of species, as well as intraspecific differences (between males and females, and between the three age groups), on the carbon and nitrogen stable isotope values were tested with parametric ANOVA, using Wilk's lambda test for significance. Differences between groups were evaluated with post hoc Tukey test.

Isotopic niches of species, as central ellipses, were calculated using SIBER (Jackson, Inger, Parnell, & Bearhop, 2011) using R ver. 3.5.0 (https://cran.r-project.org/bin/windows/base/rdevel.html) for the five most numerous small mammal species, having five or more individuals investigated for *δ* ^15^N and *δ* ^13^C. Positions of seven small mammal species, including those with sample size *n* < 5, in the isotopic biplot were shown using SigmaPlot ver. 12.5. All other calculations were performed using Statistica for Windows ver. 6.

## 3 RESULTS

### 3.1 Diversity of small mammals in the flooded meadow

Eleven species of small mammals were trapped in 2008–2016. During the investigation, the granivorous *A. agrarius* dominated the community most frequently (six out of nine years), while the herbivorous root vole (*Microtus oeconomus*) dominated in two years and the granivorous harvest mouse (*Micromys minutus*) in one year (Figure 2). In addition to these, three further species had relatively high abundance, these being the insectivorous *S. araneus* (all years, 2008–2016), the omnivorous *M. glareolus* (2014 and 2015), and the herbivorous short‐tailed vole (*Microtus agrestis*) in 2016 (Supporting information Table S1).

Diversity of the small mammal community was high (Shannon's *H* = 2.13, variation between years from the minimum of *H* = 0.67 in 2011 to the maximum *H* = 2.19 in 2015). Dominance was low, Simpson's *c* = 0.30, with a maximum in 2011 when *A. agrarius* was absolute dominant in the community, comprising 87.3% of all trapped individuals (Table 1).

An increasing dominance of *A. agrarius* was negatively related to the diversity of the small mammal community (*r* = −0.74, *n* = 9, *p* = 0.02). In the years of the strongest dominance of *A. agrarius,* the small mammal community consisted of 4–5 species, with a low abundance or absence of *M. minutus*, a species belonging to the same granivorous group (Table 1).

### 3.2 Temporal changes

Throughout the investigation, the composition of the small mammal community followed the expected numbers of species in functional groups (differences from the expected numbers were not significant). However, in five out of the nine years, the state of the small mammal community was unfavored. Favored states were found in 2011, 2013, 2015, and 2016, when numbers of species with similar dietary preferences were present according to the assembly rule (Supporting information Table S2). Unfavored states were registered when high numbers of insectivores were present in 2008, granivores in 2012, and herbivores in 2010 and 2014—that is, three functional groups had chances to be over‐rich in species.

In eight out of the nine study years, the three most abundant species were characterized by different diet preferences. Only in 2010 were two granivorous rodents *(A. agrarius, M. minutus)* dominant (Figure 2). In 2014 and 2016, years of low abundance or absence of *M. oeconomus*, *M. agrestis,* and *M. glareolus* were both recorded in high numbers. The herbivores *M. arvalis* and the water vole (*Arvicola amphibius*)*,* as well as the granivorous *A. flavicollis*, occurred in low abundances (Table 1).

Thus, based on the frequent deviations from the species assembly rule, P1 prediction was not fully confirmed, but a change in small mammal numbers over time (shifting dominance) was clearly demonstrated.

### 3.3 Isotopic partitioning

Both *δ* ^15^N and *δ* ^13^C values in *S. araneus*, *A. agrarius*, *M. oeconomus*, *M. agrestis,* and *M. glareolus* were distributed normally (Kolmogorov–Smirnov test, NS). MANOVA revealed that small mammal species had a significant effect (Hotelling's *T* ^2^ = 0.13, *p* = 0.022) on the paired *δ* ^15^N and *δ* ^13^C distribution, but not dietary group or age or sex of individuals (*T* ^2^ = 0.00, *T* ^2^ = 0.07, *T* ^2^ = 0.003, all NS, respectively). Such model explained 34% of variation of *δ* ^15^N (*r* ^2^ = 0.34, *F*_6,65_ = 7.00, *p* < 0.0001) and 48% of variation of *δ* ^13^C (*r* ^2^ = 0.48, *F*_6,65_ = 11.78, *p* < 0.0001).

Performing species‐based analysis, we found significant differences in the distribution of stable isotopes in the hair of small mammals of different species in 2015 (Wilk's lambda = 0.24, *F*_12,146_ = 12.74, *p* < 0.0001). Species had a significant effect on the differences of *δ* ^15^N and *δ* ^13^C (*F*_6,74_ = 16.64 and *F*_6,74_ = 15.38, both *p* < 0.0001).

### 3.4 Interspecific differences in dietary space

**Figure 3:**
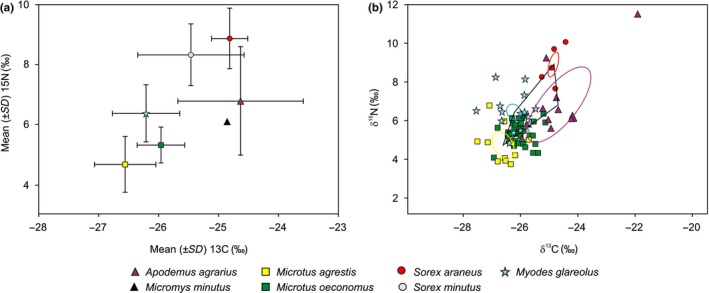
Distribution of small mammal species from the seasonally flooded meadow in Rusnė according to isotopic values (a) and central ellipses of species (b) in the isotopic space, representing fundamental niches. Bars represent 1 *SD* of the mean. Insectivorous species are shown by circles, granivorous by triangles, herbivorous by squares, and omnivorous species by stars. Central ellipses include 1 *SD* of the mean, or ~40% of data. The central ellipse of *Sorex araneus* is shown in red, *Apodemus agrarius* in magenta, *Microtus agrestis* in yellow, *Microtus oeconomus* in green, and that of *Myodes glareolus* in blue. The polygon (black line) represents the central isotopic niche of the small mammal community

The range of stable isotope values, though overlapping, showed a separation of several species (Figure 3, Supporting information Table S3) and functional groups (Supporting information Table S4). According to *δ* ^15^N, three groups were identified: the highest average isotope values being in the insectivorous shrews, with medium values in granivorous rodents (28.9% less than shrews) and the lowest values in herbivorous voles (30.2% less than granivorous mice). The omnivorous *M. glareolus* in this respect was closer to the group of granivores species (difference 5.1%).

According to *δ* ^13^C, lower values were registered in herbivorous voles and omnivorous *M. glareolus*, with higher values in shrews and mice (Figure 3a). The difference between average *δ* ^13^C values in granivores and herbivores was 5.6%, while between granivores and omnivores it was 5.9% and between omnivores and insectivores 4.3%. However, the difference between herbivores and omnivores was just 0.3% (Supporting information Table S4).

The dietary niches of the most abundant species (core ellipses in the isotopic space) were separated and did not intersect (Figure 3b), the only exception being *M. oeconomus* and *A. agrarius*, these having overlap in core ellipses of <2%. Thus, in this limited area, sympatric species of small mammals are separated dietary, confirming P2 prediction.

### 3.5 Intraspecific differences in dietary space

**Figure 4:**
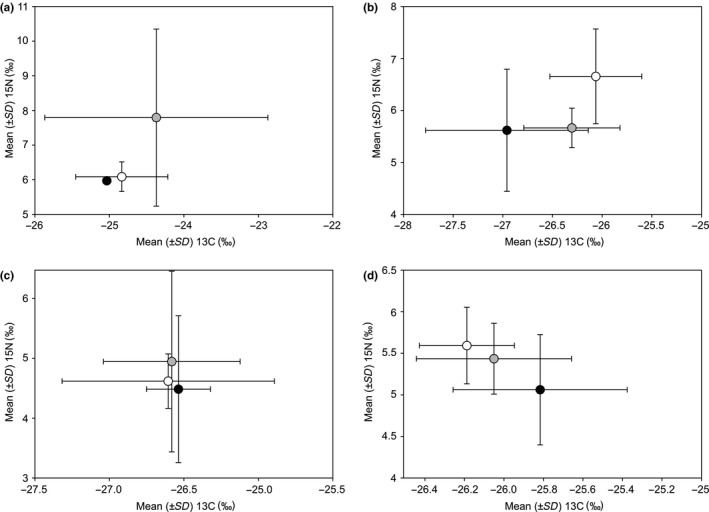
Intraspecific differences in the stable isotope values in the hair of young, subadult, and adult small mammals: a—*Apodemus agrarius*, b—*Myodes glareolus*, c—*Microtus agrestis*, d—*Microtus oeconomus*, black circles—adult, gray circles—subadult, white circles—young animals. Differences between young and adult animals in *M. oeconomus* were significant for *δ* ^13^C and had a trend for *δ* ^15^N (*p* = 0.054)

Differences in the stable isotopes in the hair of male and female small mammals were not significant in general for *δ* ^15^N (*F*_1,10_ = 1.36, *p* = 0.27) or *δ* ^13^C (*F*_1,10_ = 1.51, *p* = 0.31), nor in some separate species (Supporting information Figure S1). No significant differences between stable isotope values were found between age groups in *A. agrarius*, *M. glareolus,* and *M. agrestis* (Figure 4a–c). Thus, prediction P3 for most of the analyzed species was not confirmed. In *M. oeconomus*, differences in the stable isotopes in the hair of young, subadult, and adult small mammals were significant for *δ* ^13^C (*F*_2,31_ = 3.34, *p* = 0.048) and near‐significant for *δ* ^15^N (*F*_2,31_ = 3.21, *p* = 0.054) (Figure 4d). However, the difference expressed in percentage was not large: juveniles of *M. oeconomus* were characterized by 1.4% lower *δ* ^13^C than adult animals and 10.5% higher *δ* ^15^N.

## 4 DISCUSSION

We analyzed how several small mammal species, representing insectivores, granivores, herbivores, and omnivores, share a seasonally flooded meadow of limited size. With the re‐occupation of the habitat after the spring flood, spatial arrangement may “pack” species of small mammals tightly, high floods giving chances to uncommon species to establish (Balčiauskas et al., 2012b). We identified separation of dominant species by time and by isotopic partitioning of sympatric species, but not by intraspecific differences in diet (with one exception). Because flooded meadows are a resource‐rich habitat (Marques et al., 2015; Wijnhoven, Van Der Velde, Leuven, & Smits, 2005), unfavored small mammal community states with increased competition of several species from the same group were possible in five out of the nine years. Core areas of the three most abundant species in the isotopic space were separated, showing their dependence on different dietary resources.

### 4.1 Small mammal diversity in the flooded areas

In general, higher species diversities are characteristic of larger areas (Balčiauskas & Juškaitis, 1997), but similar patterns are also found in seasonally flooded sites. For example, in the floodplains of the Sava River, 23 small mammal species were registered (Crnobrnja‐Isailović et al., 2015), and in a much bigger area of the flooded Narewka River valley in Poland, the diversity was higher, with *H* = 2.46 and 11 species registered (Zub et al., 2012). The small mammal diversity in Rusnė did not differ from the bigger floodplains of the Vltava (*H* = 2.18, 8 species) and Danube (*H* = 2.21, 9 species) rivers (Bohdal, Navratil, & Sedlaček, 2016; Miklós, Žiak, & Hulejová, 2015).

Our diversity index (*H* = 2.13) was greater than that found in 95 out of 125 small mammal trapping sites across Lithuania, regardless of the size of these territories, which were in most cases significantly larger. Only in eight territories was the number of registered small mammal species larger than in the flooded meadow at Rusnė (re‐calculated from Balčiauskas & Juškaitis, 1997).

Of note is a new small mammal species for the Baltic countries. Mediterranean shrew (*Neomys anomalus*) was found in flooded meadows at Rusnė (<100 m from the investigated site) living sympatrically with three other shrew species, water shrew (*Neomys fodiens*), *S. araneus,* and *S. minutus* (Balčiauskas & Balčiauskienė, 2012). Two other small mammal species that are uncommon in Lithuania, namely *M. oeconomus* and *M. minutus*, may also reach high densities in the Rusnė meadows (Balčiauskas et al., 2012b). These species are not common in Lithuania (Balčiauskas & Juškaitis, 1997; Balčiauskas, 2005; Balčiauskas, Čepukienė, & Balčiauskienė, 2017 and references therein), but are not rare in flooded meadows and river valleys in other European countries (Ambros et al., 2016; Crnobrnja‐Isailović et al., 2015; Tast, 1966; Zub et al., 2012).

### 4.2 Temporal changes in the dominant species

In the flooded meadow at Rusnė, high numbers of species sympatrically shared an area of limited size. Three species dominated during the nine years of investigation: the herbivorous *M. oeconomus* during two years, the granivorous *M. minutus* in one year and the granivorous *A. agrarius* in six years. An increase in *M. oeconomus* numbers was observed every fourth year (see Table 1), while *A. agrarius* dominated in the community for the last four years of the study period (2013–2016).

It has to be noted that floods are extreme environmental phenomena, not only causing small mammal mortality, but also changing the dominant species and the resulting organization of the entire community (Thibault & Brown, 2008). Seasonal floods do not only have negative or even catastrophic effects on small mammal communities (Andersen, Wilson, Miller, & Falck, 2000), but can also influence the diversity of such communities positively (Golet et al., 2013). Generally, an increase in diversity after disturbance is observed, though such a relationship is not always linear and straightforward (Mackey & Currie, 2001). We previously found that flood height was a key factor influencing diversity and dominance in the small mammal community in the Rusnė flooded meadows. After low‐level floods, *A. agrarius* was the dominant species, while high‐level floods increased the chances for other species to dominate the meadow (Balčiauskas et al., 2012b). This corresponds to the situation described by Brown et al. (2001), where environmental perturbations can fully reorganize ecosystems, exceeding the ecological tolerances of dominant or keystone species; though changes may be buffered due to the compensatory dynamics of complementary species. In the investigated area, the 2010 flood in particular was very high (Balčiauskas et al., 2012b), and it was in this year that the two most abundant species were granivores *A. agrarius* and *M. minutus*. *A. agrarius* was shown to be the best colonizer of previously flooded areas within agricultural land (Zhang et al., 2007).

However, in an earlier (1981–1990) long‐term study of small mammals in eastern Lithuania, a different pattern of dominance was observed. In meadows, different dominant species were observed, namely *M. glareolus*, *M. arvalis,* and *S. araneus*, while *A. agrarius* numbers were always low (3.2% out of 2,346 individuals trapped) (Balčiauskas, 2005).

Thus, our recorded dominance of *A. agrarius* in the flooded meadow in six out of the nine years is not typical for Lithuania. There is no previous record of such dominance during earlier decades in various investigated habitats in the country (Balčiauskas, 2005; Balčiauskas et al., 2017; Balčiauskas & Juškaitis, 1997; Šinkūnas & Balčiauskas, 2006).

### 4.3 Diet differences and favored states

Diet differences of small mammals form the basis of their community structure. Insectivores, granivores, herbivores, and omnivores may form “favored states” if “each species entering a community will be drawn from a different functional group… until each group is represented before the cycle repeats” (Fox, 1987; Kelt, Taper, & Meserve, 1995). This pattern has been observed in different communities of small mammals (Belyea & Lancaster, 1999; Brown, Fox, & Kelt, 2000; Eccard & Ylönen, 2003; Fox & Brown, 1993; Fox & Kirkland, 1992; Kelt et al., 1995; Rodríguez & Ojeda, 2013) and in various habitats (i.e., Zub et al., 2012; Golet et al., 2013; Balestrieri et al., 2017; Ambros et al., 2016; Luza, Gonçalves, Pillar, & Hartz, 2016; Ważna, Cichocki, Bojarski, & Gabryś, 2016). Exceptions however are also known (Jánová, Heroldová, & Čepelka, 2016; Marques et al., 2015).

Several previous investigations have also confirmed favored states of small mammal communities in Lithuania (i.e., Balčiauskas & Juškaitis, 1997; Balčiauskas, 2005; Šinkūnas & Balčiauskas, 2006; Balčiauskas et al., 2017). However, in our flooded meadow, the community of small mammals was in an unfavored state (sensu Fox, 1987) in five of the nine years, and in one year, the two most numerous species, namely *A. agrarius* and *M. minutus*, were both granivores. According to Tulis et al. (2016), the negative interaction of *A. agrarius* occurs mostly with *A. flavicollis*, *M. glareolus*, *S. araneus,* and *M. minutus*. Hence, it is unusual to observe a high number of *A. agrarius* and *M. minutus* simultaneously.

One possible explanation at this locality lies in the abundance of a prevailing lush herbaceous vegetation (Wijnhoven et al., 2005) and of reed seeds at the flooded sites (Marques et al., 2015). Alternatively, the changing structure of the small mammal community as it reoccupied the vacant area after a particularly high flood may also explain this unusual co‐occurrence.

### 4.4 Isotopic partitioning

We expected that the tight packing of sympatric species and their segregation in dietary space would be reflected by stable isotope values. Testing two predictions, we found that isotopic partitioning may have helped maintain a high diversity of small mammals in the seasonally flooded meadow. Species were segregated in dietary space (confirming prediction P2), as was shown by analysis of stable isotopes from their hair. We interpret nearly full separation of the central ellipses as separation in dietary space. The only overlap in central ellipses, being less than 2%, was that between *A. agrarius* (dominant species in most years) and *M. oeconomus*.

Diversity (but not abundance) of resources in a limited area presumably should also be limited, putting constraint on the differences in *δ* ^15^N and, even more, in *δ* ^13^C values. While differences in *δ* ^15^N between insectivores, herbivores, and granivores were nearly 30%, differences in *δ* ^13^C were a mere 5%. Thus, we have to interpret dietary separation with caution, possibly because of territorial limitation.

The widest trophic niche among the small mammals was occupied by *A. agrarius*, as the variance of stable isotope values in their hair was highest (see Figure 3a and Supporting information Table S3) and the core area largest (Figure 3b). A wider trophic niche supports stability in a species (Bearhop, Adams, Waldron, Fuller, & MacLeod, 2004; Wood et al., 2017), enabling the domination of *A. agrarius* in the area of investigation. We did not find intraspecific differences of stable isotope values in most of the investigated species (prediction P3 not confirmed), with some trend in *M. oeconomus* age groups only.

Isotopic partitioning of small mammal species is characteristic in other cases of limited space, such as under snow cover (Calandra et al., 2015; Merwe & Hellgren, 2016). The segregation of the isotopic niche spaces of small mammals, minimizing interspecific competition, allows sympatric species to coexist (Baltensperger et al., 2015), especially in grasslands, where small mammals are more plastic in their dietary preferences (Symes et al., 2013).

However, we found no other studies for comparison with regard to isotopic partitioning in small mammal species in a small area equivalent to the Rusnė flooded meadow. Although intraspecific dietary separation was found in *A. flavicollis* and *M. glareolus* living in the territory of a great cormorant colony, we interpret this as competition for scarce food resources and as adults feeding in the best habitats (Balčiauskas et al., 2016 and references therein). We suppose that abundant and diverse food in the cyclic habitat of the flooded meadow allows most species to avoid intraspecific competition. As for *M. oeconomus*, it is a relatively new species in Lithuania, arriving only about half a century ago (Balčiauskas, Balčiauskienė, & Baltrūnaitė, 2010), and thus, it may have a different strategy of habitat use.

## 5 CONCLUSIONS AND SIGNIFICANCE

We found that the small mammal community in the restricted area of flooded meadow maintained a high species diversity despite a cyclic stressor (flood) due to isotopic partitioning (segregation in dietary space) and by changes in their number over time (shifting dominance). The shifting of dominant species maintains long‐term diversity, reflecting the differing resilience of various species to the floods, while separation in dietary space most probably only works at the level of the current year. In most years, the three most abundant species represented each of the different functional groups, insectivores, granivores, herbivores, and omnivores. However, in five of the nine years, the community was in an unfavored state. Segregation of species in dietary space was confirmed by stable isotopes from their hair, with the only overlap in central ellipses occurring between *A. agrarius* (dominant in most years) and *M. oeconomus*. The dominant species, *A. agrarius*, was characterized by the widest diet.

In the future, with respect to climate change and the resultant expected increases in extreme flood events in northern Europe (Reader et al., 2014) and the arrival of new species due to changes in distribution ranges and consequent changes to communities and food webs (Baltensperger et al., 2015), knowledge of the formation of small mammal communities may help in the prognosis of ecosystem changes and predicting at‐risk species.

## CONFLICT OF INTEREST

None declared.

## AUTHORS' CONTRIBUTIONS

LB1 formulated the research idea, did statistical analysis, and drafted the manuscript; LB1, LB2, and MJ trapped small mammals; LB2 identified species, performed literature overview, and revised all manuscript versions; RS and MJ performed stable isotope analysis. All authors contributed critically to the drafts and gave final approval for publication.

## Data Availability

Data from this study (stable isotope raw data matrix) available from the Dryad Digital Repository: https://doi.org/10.5061/dryad.2rc8s7m.
