# Supplementary material for: Resource partitioning confirmed by isotopic signatures allows small mammals to share seasonally flooded meadows
Source: Ecol Evol. 2019 Apr 8;9(9):5479–89. doi: 10.1002/ece3.5144 (PMC6509440; doi:10.1002/ece3.5144)
Supplement: Supplementary file 2 [file ECE3-9-5479-s002.doc]

**Supporting information**

**Figure S1** Intraspecific differences of the stable isotope values in the hair of males and females:

A – *Apodemus agrarius*, B – *Myodes* *glareolus*, C – *Microtus* *agrestis*, D – *Microtus* *oeconomus*, black circles – males, white circles – females. None of the differences were significant

**Table S1** Relative abundance of the small mammal community in a seasonally flooded meadow at Rusnė (western Lithuania), 2008–2016, standardized to 100 trap nights. Diet preferences marked with superscripts: a – insectivores, b – granivores, c – herbivores, d – omnivores (according Butet & Deletre, 2011; Churchfield & Rychlik, 2006; Zub, Jędrzejewska, Jędrzejewski, & Bartoń, 2012; Pernetta, 1976).

| Species | 2008 | 2009 | 2010 | 2011 | 2012 | 2013 | 2014 | 2015 | 2016 |
| --- | --- | --- | --- | --- | --- | --- | --- | --- | --- |
|
| *Sorex araneus* ^a^ | 4.67 | 2.11 | 2.03 | 0.33 | 5.87 | 2.00 | 2.06 | 2.00 | 1.56 |
| *Sorex minutus* ^a^ | 0.40 | 0.40 | 0.79 | – | 0.40 | 0.44 | – | 0.89 | 0.67 |
| *Neomys fodiens* ^a^ | 0.27 | – | – | – | – | – | – | – | – |
| *Apodemus agrarius* ^b^ | 2.93 | 3.01 | 12.66 | 20.67 | 7.60 | 3.78 | 8.08 | 11.78 | 14.00 |
| *Apodemus flavicollis* ^b^ | – | – | – | – | 0.40 | – | – | – | – |
| *Micromys minutus* ^b^ | 7.07 | – | 2.16 | – | 1.33 | – | 0.73 | 0.44 | 0.22 |
| *Microtus arvalis* ^c^ | – | 0.10 | – | – | – | – | 0.29 | – | – |
| *Microtus agrestis* ^c^ | – | – | 0.07 | – | – | – | 0.44 | 2.22 | 1.56 |
| *Microtus oeconomus* ^c^ | 6.13 | 5.11 | 1.97 | 2.33 | 8.93 | 1.11 | 1.03 | 8.22 | – |
| *Myodes glareolus* ^d^ | – | – | 0.85 | 0.33 | 0.27 | 0.22 | 1.47 | 2.89 | 0.67 |
| *Arvicola amphibius* ^c^ | – | – | 0.07 | – | – | – | – | – | – |
| Total, N | 21.47 | 10.73 | 20.59 | 23.67 | 24.80 | 7.56 | 14.10 | 28.44 | 18.67 |

**Table S2** Distribution of the favored and unfavored states of small mammal communities in Rusnė flooded meadow, based on the number of species in each functional group in 2008–2016. Years of favored states shown in bold. In all years differences of the number of species in functional groups were not significant from expected distribution

| Year | Number of species in the functional group of | | | |
| --- | --- | --- | --- | --- |
|  | insectivores | granivores | omnivores | herbivores |
| 2008 | 3 | 2 | 0 | 1 |
| 2009 | 2 | 1 | 0 | 2 |
| 2010 | 2 | 2 | 1 | 3 |
| **2011** | **1** | **1** | **1** | **1** |
| 2012 | 2 | 3 | 1 | 1 |
| **2013** | **2** | **1** | **1** | **1** |
| 2014 | 1 | 2 | 1 | 3 |
| **2015** | **2** | **2** | **1** | **2** |
| **2016** | **2** | **2** | **1** | **1** |

**Table S3** Range of *δ*^15^N and *δ*^13^C values in the hair of small mammals from the seasonally flooded meadow at Rusnė and significance of inter-species differences. Diet preferences are marked with superscripts: a – insectivores, b – granivores, c – herbivores, d – omnivores. Different superscript numbers show significant differences of average *δ*^15^N and *δ*^13^C values according to Tukey‘s HSD (p < 0.05)

| Species | N | *δ*^15^N_min_ | *δ*^15^N_max_ | *δ*^13^C_min_ | *δ*^13^C_max_ |
| --- | --- | --- | --- | --- | --- |
| *Sorex araneus* ^a^ | 5 | 7.62^1^ | 10.05 | -25.27^1^ | -24.43 |
| *Sorex minutus* ^a^ | 3 | 7.23^12^ | 9.27 | -26.38^1^ | -24.64 |
| *Apodemus agrarius* ^b^ | 12 | 4.98^2^ | 11.46 | -25.92^1^ | -21.93 |
| *Micromys minutus* ^b^ | 1 | 6.07 |  | -24.89 |  |
| *Microtus agrestis* ^c^ | 11 | 3.67^3^ | 6.71 | -27.50^2^ | -25.67 |
| *Microtus oeconomus* ^c^ | 34 | 4.05^3^ | 6.28 | -26.90^2^ | -25.14 |
| *Myodes glareolus* ^d^ | 15 | 4.78^24^ | 8.21 | -27.53^2^ | -25.46 |

**Table S4** Average (±SE) and range of *δ*^15^N and *δ*^13^C values in the hair of small mammal functional groups from the seasonally flooded meadow at Rusnė and significance of inter-group differences. Different superscript numbers show significant differences of average *δ*^15^N and *δ*^13^C values according to Tukey‘s HSD (p < 0.05)

| Group | N | *δ*^15^N_avg±SE_ | *δ*^15^N_min_ | *δ*^15^N_max_ | *δ*^13^C_avg±SE_ | *δ*^13^C_min_ | *δ*^13^C_max_ |
| --- | --- | --- | --- | --- | --- | --- | --- |
| Insectivores | 5 | 8.66±0.35^1^ | 7.23 | 10.05 | -25.08±0.22^1^ | -26.38 | -24.43 |
| Granivores | 13 | 6.72±0.48^2^ | 4.98 | 11.46 | -24.68±0.27^1^ | -25.92 | -21.93 |
| Herbivores | 34 | 5.16±0.11^3^ | 3.67 | 6.71 | -26.14±0.07^2^ | -27.50 | -25.14 |
| Omnivores | 15 | 6.38±0.25^2^ | 4.78 | 8.21 | -26.22±0.14^2^ | -27.53 | -25.46 |
